# The role of the metabolite cargo of extracellular vesicles in tumor progression

**DOI:** 10.1007/s10555-021-10014-2

**Published:** 2021-12-27

**Authors:** Mária Harmati, Mátyás Bukva, Tímea Böröczky, Krisztina Buzás, Edina Gyukity-Sebestyén

**Affiliations:** 1 Laboratory of Microscopic Image Analysis and Machine Learning, Institute of Biochemistry, Biological Research Centre – Eötvös Loránd Research Network, 6726 Szeged, Hungary; 2 Department of Immunology, University of Szeged, 6720 Szeged, Hungary; 3 Doctoral School of Interdisciplinary Medicine, University of Szeged, 6720 Szeged, Hungary

**Keywords:** Extracellular vesicles; Metabolites, Cancer, Metastasis

## Abstract

Metabolomic reprogramming in tumor and stroma cells is a hallmark of cancer but understanding its effects on the metabolite composition and function of tumor-derived extracellular vesicles (EVs) is still in its infancy. EVs are membrane-bound sacs with a complex molecular composition secreted by all living cells. They are key mediators of intercellular communication both in normal and pathological conditions and play a crucial role in tumor development. Although lipids are major components of EVs, most of the EV cargo studies have targeted proteins and nucleic acids. The potential of the EV metabolome as a source for biomarker discovery has gained recognition recently, but knowledge on the biological activity of tumor EV metabolites still remains limited. Therefore, we aimed (i) to compile the list of metabolites identified in tumor EVs isolated from either clinical specimens or *in vitro* samples and (ii) describe their role in tumor progression through literature search and pathway analysis.

## Introduction

Under normal and pathological conditions, most cells secrete a range of membrane-bound extracellular vesicles (EVs). Although their physical characteristics overlap, EVs are highly heterogeneous, and several subpopulations have been described. Microvesicles and exosomes are primary subtypes of EVs differentiated by their biogenesis, release pathway, size, content, and function [1, 2].

Initially, EV secretion was thought to be a cellular waste disposal mechanism. Since then, it has been clearly demonstrated that EVs play a key role in intercellular communication by mediating horizontal transfer of diverse molecular content between adjacent and distal cells [3–5]. These delivery vehicles are excellently equipped to protect their cargo inside the lipid bilayer from extracellular enzymes, and they are able to cross different biological barriers, such as the blood–brain barrier [6, 7]. There is also accumulating evidence that they fulfill the two main criteria of the EV-mediated communication: (i) selective packaging of signaling content into the newly formed vesicles, and (ii) selective delivery of EVs to target cells [2].

Recently, it has been recognized that metabolite content of EVs may have a prominent role in EV-mediated communication in tumor diseases. Despite the technical challenges of EV metabolite analysis, investigation of the EV metabolite cargo, its role in tumor progression, and potential in clinical diagnosis deserve further attention.

In this review, we provide insight into EV biology and the technical aspects of EV studies. We also describe the current knowledge on the functional role of the EV-transferred metabolites in tumor progression.

## Tumor EV biology and research

### Biogenesis of EVs

**Fig. 1:**
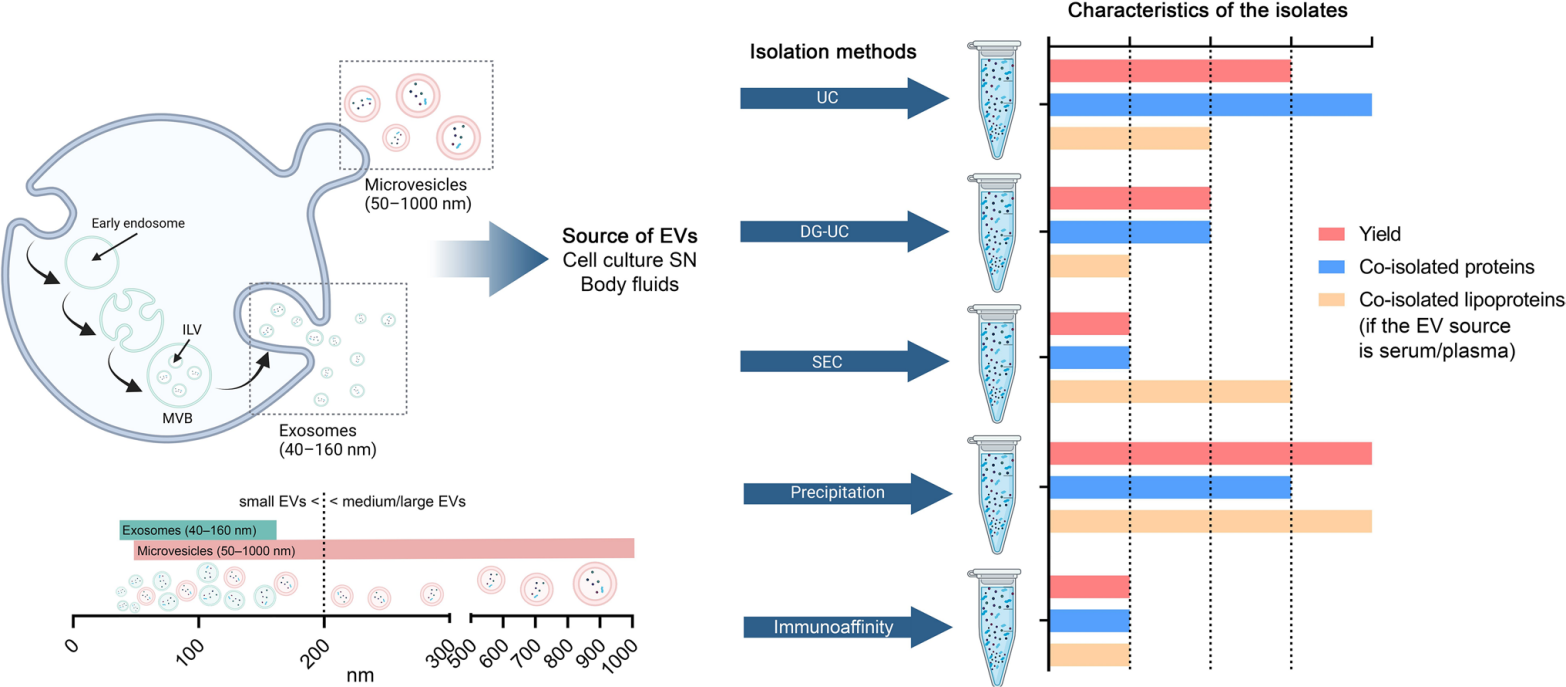
Biogenesis and isolation methods of EVs. The left side of the figure shows a schematic overview of the main EV biogenesis pathways. The bottom left of the figure shows how EVs are classified by biogenesis (exosomes and microvesicles) and by size (small and medium/large EVs), indicating the overlap in the size range of the different EV types. The right side of the figure shows the main isolation methods and the comparison of their most important indicators such as yield, and co-isolated contaminants [10, 11]. Abbreviations: ILV, intraluminal vesicle; MVB, multivesicular body; EVs, extracellular vesicles; SN, supernatant; UC, ultracentrifugation; DG-UC, density gradient-ultracentrifugation; SEC, size-exclusion chromatography. This figure was created at BioRender.com

Based on their biogenesis pathways, EVs are divided into two major classes—ectosomes (or microvesicles) and exosomes [5, 8, 9] (Fig. 1). The membrane budding step, a common feature in this pathway, is similar in both classes. In addition, both EV types bud away from the cytoplasm resulting in the same membrane orientation, which is identical to the orientation of the plasma membrane [12]. In the case of ectosomes, this budding step occurs outward at the plasma membrane and results in a direct release of EVs ranging from ~ 50 nm to 1 μm in diameter. In contrast, exosomes are formed as intraluminal vesicles (ILVs) through inward budding of endosomes, which develop into multivesicular bodies (MVBs). These MVBs may fuse with lysosomes for degradation or fuse with the plasma membrane resulting in the extracellular release of ILVs as exosomes (40–160 nm in diameter) [8]. The precise molecular mechanisms of EV biogenesis have only recently started to be understood. The main driver of exosomal biogenesis is the endosomal sorting complex required for transport (ESCRT), but the existence of ESCRT-independent routes has also been proven [4, 13]. Despite their different biogenesis routes, intracellular mechanisms and sorting machineries of ectosomes and exosomes partially overlap. Shared features of different EVs make it difficult to distinguish between vesicle subpopulations [14].

### Composition of EVs

EVs convey numerous proteins (e.g., tetraspanins, chaperones, biogenesis factors, signaling molecules), nucleic acids (e.g., miRNA and other non-coding RNAs, mRNA, DNA), small metabolites (e.g., sugars, amino acids, vitamins), and lipids (e.g., phosphatidylserine, cholesterol, ceramide), which are all selectively packed into vesicles in a cell type-dependent manner [14]. As EVs are distinct entities of the complex intercellular communication, their molecular fingerprint depends on the quality and state of the donor cell, and it is often influenced by microenvironmental stimuli [5, 15, 16].

### EV signaling and uptake mechanisms

The mechanisms EVs used to interact with the cell surface and transfer their cargo into the target (recipient) cells are not fully understood. Literature data suggest that these mechanisms depend on the origin and type of EV as well as the target cell [14, 17].

EVs may induce a phenotypic response in the recipient cell without internalization; receptor–ligand interactions may be sufficient to elicit signal transduction. Alternatively, EVs may transfer their cargo by direct fusion with the plasma membrane, and they may also be internalized via an active endocytic process, i.e., clathrin-, caveolin-, and lipid raft-mediated endocytosis, macropinocytosis, or phagocytosis [18]. Once in the cell, intraluminal EVs may fuse with the endosomal limiting membrane to release their content into the cytoplasm and elicit phenotypic responses in the recipient cell [14, 18, 19].

### EV terminology

Since the origin and the physical and functional characteristics of EVs are diverse, several terms have been used for EVs in the literature. The prefixes micro- and nano- refer to their size (microvesicles, microparticles, nanovesicles, nanoparticles); ecto- and exo- refer to their presence outside the cells (ectosomes, exosomes, exovesicles). Other terms, such as oncosomes and tolerosomes, indicate their origin or function, respectively [13]. Although the nomenclature is continuously evolving, the International Society for Extracellular Vesicles (ISEV) recommends the use of “extracellular vesicle” as the “generic term for particles naturally released from the cell that is delimited by a lipid bilayer and cannot replicate.” They also suggest the use of operational terms for EV subtypes that refer to their (i) physical characteristics (small or medium/large EVs), (ii) biochemical composition (CD81^+^ EVs), or (iii) conditions of release (hypoxic EVs) [1, 20]. Here, we use the terms found in the referenced articles.

### Role of EVs in cancer

Exosomes and other classes of EVs are important mediators of cell–cell communication and play an essential role in cancer biology. It has long been well known that cancer cells secrete higher amounts of EVs than healthy cells. EVs in higher numbers have been detected in the plasma of cancer patients as well as in tumor cell cultures [21, 22].

**Fig. 2:**
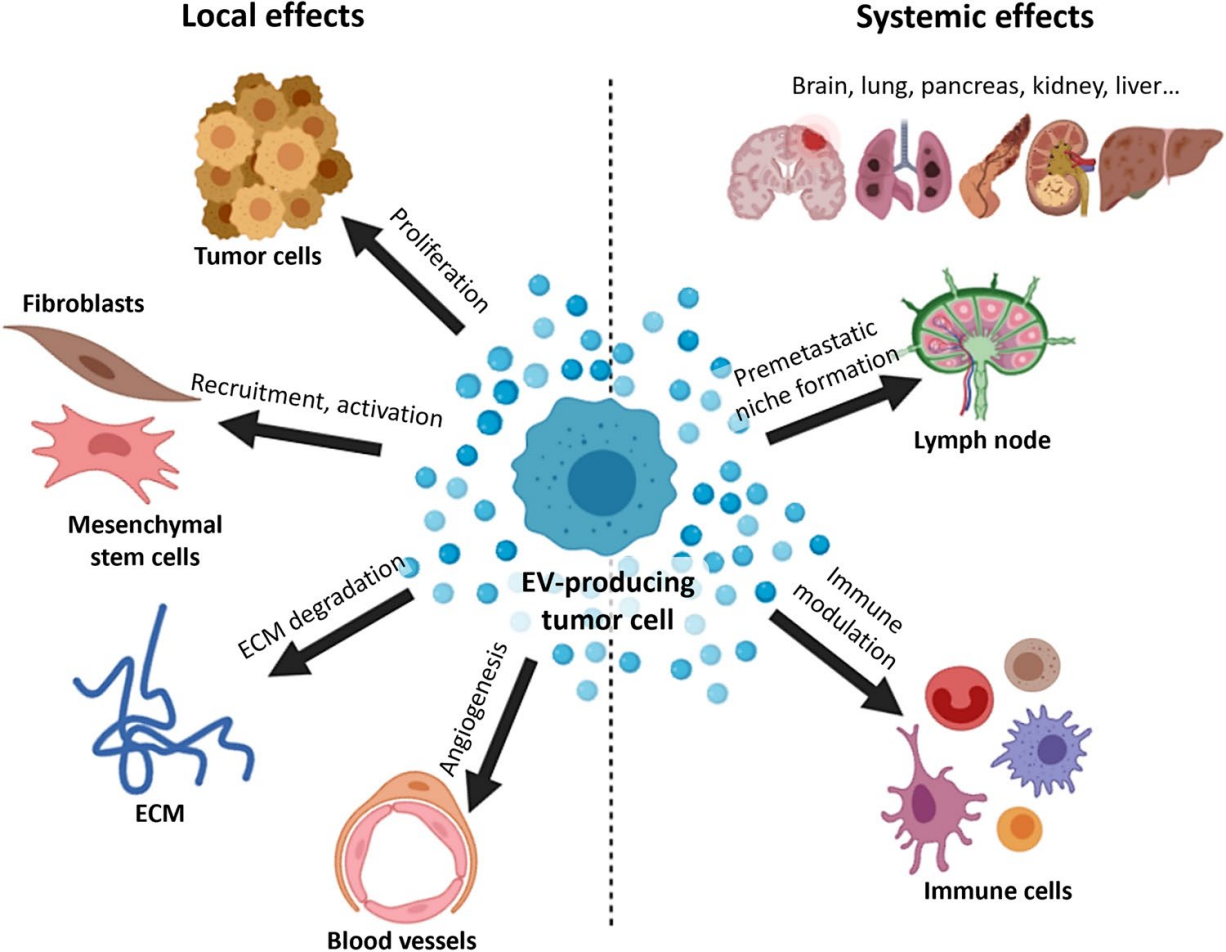
Tumor-derived EVs have both local and systemic effects. These EVs can alter the TME, modulate immune responses and prepare distant tissue sites for metastasis. This figure shows some examples of the tumor EV effects. Abbreviation: ECM, extracellular matrix; the figure was created based on [28] at BioRender.com

Exosomes contribute substantially to tumor progression, invasion, and metastasis by horizontally transmitting a variety of surface and signaling molecules, oncogenic proteins, and nucleic acids to target cells, thereby altering their behavior [23, 24]. For instance, locally, in the tumor microenvironment (TME), tumor-derived EVs may convey resistance to neighboring tumor cells. These EVs can also re-educate fibroblasts and mesenchymal stem cells or activate endothelial cells, thereby inducing angiogenesis. Systemically, EVs have a crucial role in immune modulation and pre-metastatic niche formation [7, 25–28] (Fig. 2). However, communication is not unidirectional in tumors. On the contrary, a complex, systemic communication network develops in parallel with the tumor evolution [29].

As mentioned above, the characteristic molecular fingerprint of small EVs (sEV), i.e., exosomes, is not independent of the parent/donor cell status. The metabolic status of cancer cells influences exosome secretion and content. Hypoxia, starvation, and acidosis are among the typical metabolic conditions that cancer cells undergo in the TME. Notably, all of these conditions have been shown to influence not only the rate of exosome secretion, but also the molecular composition of exosomes [30].

### EV isolation and characterization methods

Before launching any investigation, one must consider the complexities of working with EVs. For instance, preparations obtained using isolation procedures that target exosomes may contain other EVs as contaminants due to the overlapping physical and biomolecular features of exosomes and microvesicles. Research guidelines for EVs are provided in the Minimal Information for Studies of Extracellular Vesicles (MISEV) to support the transparency and reproducibility of EV studies [1].

A standardized method for the isolation of EVs from cell culture supernatants or body fluids (blood, urine, saliva, etc.) has not yet been established. There are several alternative approaches to isolate and purify EVs. Important factors to consider when choosing a method include the type and volume of the EV source, the target EV subtype, the target EV yield and purity of isolates, and the downstream metabolite analysis technique. Depending on the isolation method and the EV source, abundant serum proteins (albumin, globulins) and various lipoproteins (chylomicrons, HDL, LDL, and VLDL) as well as nucleic acids on the surface of EVs may contaminate the EV isolates due to their similar physical properties. These co-isolated contaminants may interfere with the particle number and size distribution measurements and mislead biomarker analyses or functional assays [10].

Previous research has shown that EV subgroups have a unique biochemistry and function [31, 32]. This is consistent with the results of Luo et al. who found that different types of vesicles from pleural effusions showed unique metabolic enrichments [33]. Isolation methods themselves may also modify EV composition. In prostate cancer cell line models, the metabolic signature varies according to the conditions of cell culture [34]. Due to the broad range of physical and chemical characteristics of distinct metabolites, it is hardly possible to quantify all metabolites using a single approach. Therefore, it is necessary to select an appropriate method for metabolite analysis. Gas chromatography-mass spectrometry (GC–MS), liquid chromatography-mass spectrometry (LC–MS), and capillary electrophoresis-mass spectrometry (CE-MS) are the most common mass spectrometric methods used for metabolomic analyses. Additionally, in recent years, improved analytical techniques have emerged, such as ion chromatography-mass spectrometry (IC-MS) to analyze highly hydrophilic compounds [35, 36] or the capillary IC-MS as a selective and specific method to analyze anionic metabolites [37], e.g., nucleotides, sugar phosphates, and organic acids. Williams and colleagues have collected and highlighted several practical pitfalls in the field of EV metabolomics research [38].

### EV metabolomics

Although lipids are dominant components of EVs, the vast majority of the EV cargo studies have investigated the protein and nucleic acid content of EVs; only a few researches have analyzed the lipid or small metabolite composition [38]. In line with this observation, EV databases, such as Vesiclepedia, ExoCarta, EVpedia, or miREV, mainly contain protein, mRNA, and miRNA entries with less lipid and metabolite data [39–42].

The composition of EVs is comparable to that of the source donor cells, but they are also enriched in certain lipids such as cholesterol, phosphatidylserine (PS), phosphatidylcholine (PC), and phosphatidylinositol (PI), suggesting that EV may operate as cell-to-cell lipid mediators [43]. From a practical and clinical standpoint, studying the metabolomics of EVs isolated from human biofluids is the most suited approach, since the metabolome of these vesicles contains a goldmine of disease biomarkers. The EV metabolome’s potential as a source for biomarkers was first demonstrated by comparative metabolomics of plasma-derived EVs from endometrial cancer patients and healthy controls, which revealed valuable differences in these two groups [44].

## Functional role of EV-transferred metabolites in cancer

**Fig. 3:**
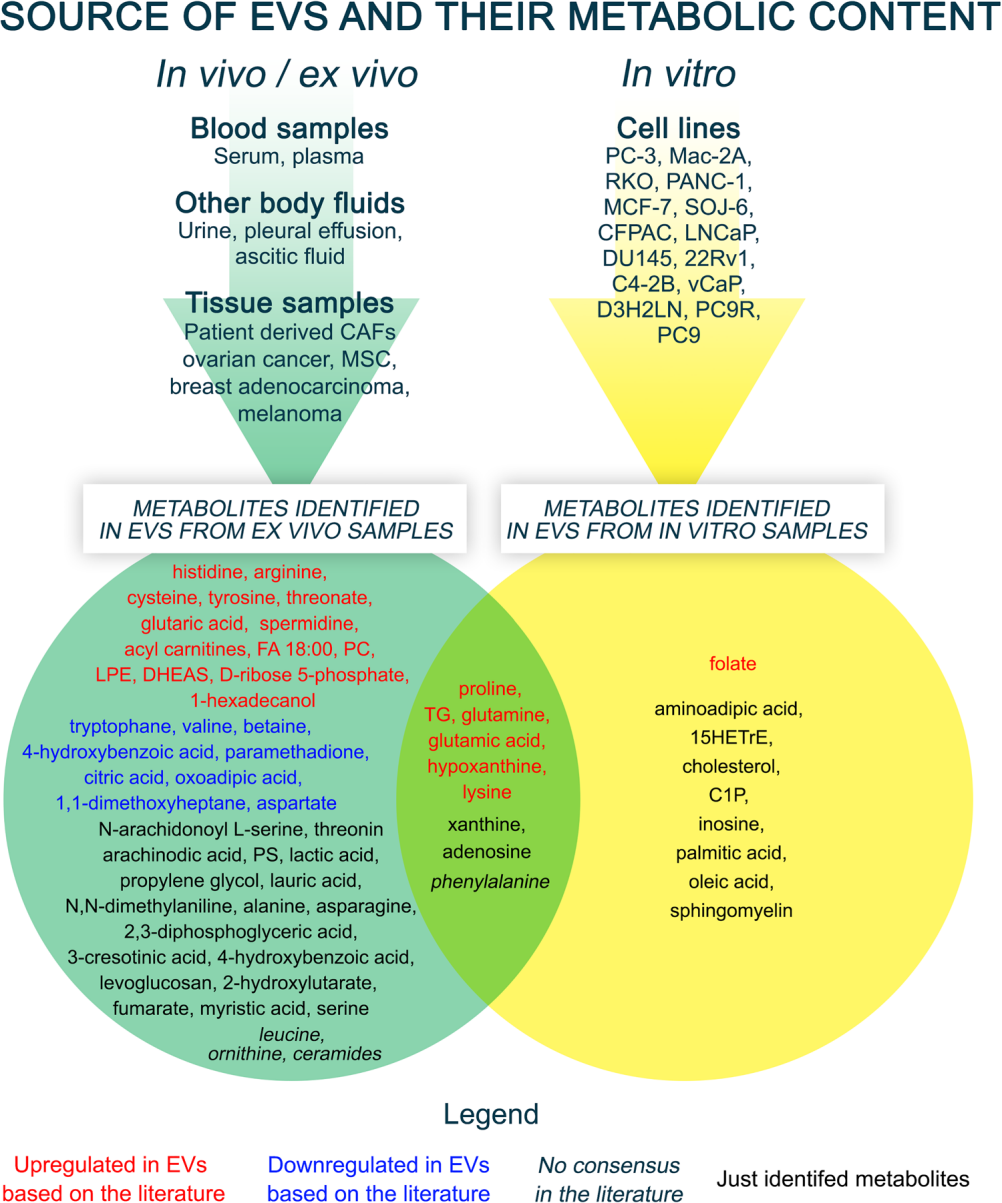
Summary Venn diagram of EV metabolites. The figure summarizes the metabolites identified in the literature according to their source and expression. The top of the figure shows the sources of the EVs investigated in the different studies, and the identified metabolites are shown at the bottom. The different colors and font styles indicate the expression state of metabolites. The figure was created using GIMP

Numerous studies have shown that the tumor- and tumor stroma-derived EVs alter the metabolism of the recipient cells. Several studies highlight the differences in the metabolite profile of EVs between diseased and normal states as well as between various stages of tumors and/or suggest biomarkers for diagnosis, prognosis, or treatment schedule choice [45, 46]. At the same time, the knowledge on the biological activity of tumor EV metabolites remains limited. The primary aim of this review is to collect this knowledge focusing on the role of EV metabolites in tumor progression. We collected a list of metabolites identified in tumor EVs isolated from either clinical specimens or *in vitro* samples (Fig. 3) and describe their functional effects according to main metabolite types.

### Amino acids, amines, and their derivatives

#### Amino acids

Amino acids (AA) present in the metabolome of EVs have been investigated in both *in vitro* and *ex vivo* experiments. In these studies, both cell culture supernatants and patient-derived biofluids such as urine, serum, or plasma have been used as sources of EVs.

Recent findings indicate that the AA content of EVs secreted by the cells may be a source of nutrients for the recipient cells by entering into different metabolic pathways or by acting on cell motility and proliferation through other pathways. The results of Onozato et al. revealed that certain AAs—histidine, arginine, glutamine, cysteine, lysine, and tyrosine—are significantly enriched in the exosome-eluted fraction from healthy human serum, but no functional analyses were performed [47].

Numerous studies have reported the increased expression of AAs or their derivatives in tumors, but so far, no clear consensus on a shared set of AAs across various malignancies has been achieved. Palviainen and colleagues observed that proline was upregulated in all EVs derived from prostate cancer (PCa), cutaneous T-cell lymphoma (CTCL), and colon cancer (CC) cell lines (PC3, Mac-2A, RKO) when compared to their respective controls [48]. Proline is a unique AA that plays a key function not only in protein biosynthesis but also in cancer metabolism as a regulatory AA. Altered proline biosynthesis in tumor tissue leads to increased proliferation and biomass production [49, 50]. During the degradation of proline, the p53 gene-induced proline dehydrogenase/proline oxidase pathway produces adenosine triphosphate (ATP) for autophagy and reactive oxygen species (ROS) for apoptosis [51]. Surazynski et al. have shown that proline can inhibit the degradation of hypoxia-inducible factor-α (HIF-1α) via the von Hippel-Lindau protein-dependent proteasomal pathway [52]. HIF-mediated pathways have a significant impact on metabolic response, erythropoiesis, angiogenesis and vascular tone, cell proliferation and differentiation, survival, and apoptosis; thus, they are crucial factors in cancer [53].

Luo and colleagues compared the metabolic profile of large EVs (lEVs) and sEVs in malignancy pleural effusion (MPE) and tuberculosis pleural effusion (TPE) samples [33]. In the lEV samples, more AAs were decreased in MPE, such as phenylalanine, tryptophan, leucine, valine, ornithine, and betaine; in contrast, threonate and glutaric acid were elevated in the MPE lEV samples. Luo and colleagues have identified a relationship between these metabolite variations in lEVs and biological and clinical parameters. The levels of carcinoembryonic antigen (CEA) and pleural adenosine deaminase show significant correlations with different AA levels in lEVs, but this correlation was moderate in sEVs [33]. Aspartate, a metabolite that plays an important role in protein synthesis and is a precursor of cell signaling molecules, has been found in MPE EVs [33].

Altadill et al. have identified significant amounts of AAs and AA derivatives in sEVs isolated from the supernatants of the PANC1 human pancreatic carcinoma cell line [44]. Although they did not perform functional assays, the molecules identified have previously been shown to be involved in tumor development and metabolic pathways. Aminoadipic acid is a well-known intermediate in the synthesis of acetyl-CoA; therefore, it is closely linked to the tricarboxylic acid (TCA) cycle and cellular energy balance [54]. Aminoadipic acid plays a role in the synthesis of lysine, various modifications of which may contribute to tumor development through several metabolic pathways [55]. Aminoadipic acid is also known to have direct effects on various cells, such as enhancing glial cell migration and glioblastoma aggressiveness [56, 57].

Other studies have also pointed to the involvement of the TCA cycle. Palviainen et al. investigated the effect of the biochemical composition of lEVs and sEVs isolated from supernatants of two prostate cancer cell lines (PCa, VCaP) in silico and found that AAs present in vesicles mainly affect the TCA cycle, thereby providing energy to fuel the intensive metabolism of the rapidly dividing recipient tumor cells for [34]. Zhao et al. have shown that cancer-associated fibroblasts (CAFs) secrete exosomes to regulate the metabolism of recipient cancer cells [58]. They detected particularly high levels of glutamine, arginine, glutamate, proline, alanine, threonine, serine, asparagine, valine, and leucine in prostate CAF-derived exosomes (CDEs). Additionally, in pancreatic CDEs, they found high levels of glutamine, threonine, phenylalanine, valine, isoleucine, glycine, arginine, and serine [58]. Zhao et al. provided a compelling proof-of-concept that AAs in CDEs can supply TCA cycle metabolites to cancer cells under both complete and nutrient-deprived conditions. Using isotope tracing, they demonstrated that these metabolites are used as precursor metabolites by the recipient cancer cells for proliferation and also to restore the levels of the TCA cycle metabolites [58].

Puhka et al. isolated lEVs and sEVs from serum and urine samples of healthy volunteers and PCa patients and detected a high concentration of ornithine in PCa urine and plasma EVs in contrast to healthy EVs [59]. Their results emphasize the importance of the non-proteinogenic AA ornithine in addition to the proteinogenic AAs discussed above. Ornithine has previously been described as an important precursor of polyamines, which show elevated levels during carcinogenesis [59]. Gökmen et al. found that ornithine levels can be useful to distinguish patients with malignant skin tumors from healthy subjects [60].

Vallabhaneni et al. have directly investigated the effect of sEVs secreted by patient-derived mesenchymal stem cells on MCF-7 breast tumor mouse xenograft models [61]. Their findings showed that sEV treatment accelerated tumor growth compared to the control group. They hypothesized that—among other factors—the high concentrations of glutamic acid determined in sEVs may enhance cell proliferation, as glutamine can not only contribute to the TCA cycle but can also serve as a carbon and nitrogen source for all major macromolecules [61].

#### Amines

In addition to ornithine, Puhka et al. found elevated levels of an aliphatic polyamine called spermidine in PCa EVs. The high amount of spermidine may be caused by the high activity of the enzyme ornithine decarboxylase, the rate-limiting enzyme in the polyamine synthase pathway [59]. Various studies have shown that polyamine biosynthesis is upregulated in actively growing cells, including cancer cells. The elevated level of polyamines in the TME has a role in cancer cell transmigration into the circulation leading to metastasis formation and helps cancer cells escape recognition by the immune system [62]. N,N-Dimethylaniline, a member of the amines group, has been detected in serum-derived exosomes from head and neck cancer (HNC) patients [45].

#### Derivatives

Clos-Garcia et al. found increased levels of acylcarnitines (the acetylated forms of L-carnitine) in the urinary EVs from PCa patients [63]. Puhka and coworkers previously suggested that variable carnitine levels in PCa EVs correlated with a metabolic shift towards β-oxidation of fatty acids (FA) [59]. Altadill et al. have shown the presence of N-arachidonyl L-serine in plasma exosome-like vesicles (ELVs) obtained from patients with endometrioid adenocarcinoma [44]. N-arachidonyl L-serine has been found to promote cell migration, proliferation, and angiogenesis [64].

### Lipids

Lipids are a main class of biological compounds with a wide variety of structural and signaling roles. Apart from sterols, most lipids have hydrophobic side chains and polar head groups, and the rich lipid diversity is the result of different combinations of side chains and head groups. Despite their comparable molecular complexity, there is a better understanding of the function of proteins than that of lipids, which are called as the “Cinderellas” of cell biology by Muro et al. [65].

Exosomes predominantly contain lipids, including diglycerides, sphingolipids, phospholipids, and phosphoglycerolipids, and they are enriched in specific lipids, such as cholesterol, PS, PC, and PI, which may function as cell-to-cell lipid mediators [43]. Exosomes may also carry specific bioactive lipids, including leukotrienes and prostaglandins [66].

There is accumulating evidence that the lipid content of EVs and parental cells differ; for instance, elevated levels of diacylglycerols (DAG), ceramides (Cer), sphingomyelin (SM), PC, phosphatidylethanolamines (PE) and FA were detected in EVs [31, 67, 68]. In line with this, Luo et al. identified phosphoglycerolipids, sphingolipids, and glycerolipids as major differential lipid species in lEVs and sEVs when comparing TPE with MPE enrichment of specific lipid metabolites in EVs may affect the cellular function of target cells and reflect the metabolic state of parent cells [33].

Lipids have a key role in the production and biological functions of EVs [33]. Sphingolipids, such as Cer, are critical not only in the formation and release of EVs [69], but in the regulation of cell survival and inflammation as well [70]. SM, PS, PC, PI, and cholesterol may occur in four times higher amounts in EVs than in parental cells, which contributes to the increased membrane rigidity of exosomes, and their role in the recognition and internalization of exosomes [43].

#### Fatty acids

Paolino et al. showed that FA and protein compositions of plasma-derived sEVs from stage 0–I, II, and III–IV melanoma patients could reflect disease stages. FA analysis of CD81-expressing sEVs (CD81sEVs) revealed that several FA species are more abundant in EVs obtained from cancer patients than those from healthy donors. They also discussed the role of these FAs in disease progression [46]. For instance, higher levels of lauric (C12:0) and myristic (C14:0) acids in stage II and III–IV CD81sEVs may result from the accelerated metabolism of advanced cancer [71]; also, higher oleic acid (C18:1) levels in stage II and III–IV may increase membrane fluidity supporting the adhesion and migration, since the correlation between C18:1 and the metastatic potential has already been established [72].

Elevated levels of saturated FAs (FA 18:0) were observed in MPE EVs compared to EVs in TPE [33], and FAs can also be used to provide energy through β-oxidation and accelerate lung tumorigenesis [73].

Wojakowska and colleagues detected heptanoic acid in serum exosomes, but not in whole serum from HNC cancer patients. However, there were no significant differences in heptanoic acid levels of the vesicles between the healthy controls and pre-treated and post-treated cancer samples [45].

According to Schlaepfer et al., EVs may support growth following reoxygenation in a survival response of prostate cancer cells to hypoxic stress. Palmitic and oleic FAs transferred by hypoxic EVs may serve a dual purpose; they can be used for membrane synthesis and ATP generation as they are built into phospholipids or utilized as fuel in the mitochondria in the oxygenated recipient cells in the periphery of the tumors. This way, hypoxic PCa EVs may contribute to the overall aggressiveness of the tumor [74].

Other FAs in EVs, e.g., arachidonic acid (Aa), can also be delivered to intracellular membrane-localized enzymes, which enable bioactive lipid generation and stimulate growth and motility of the target cells [74]. Indeed, Aa is the precursor of important proliferative and inflammatory modulators, e.g., eicosanoids and prostaglandins [63]. However, Clos-Garcia et al. found reduced levels of Aa in PCa urine EVs [75]. They hypothesized that the increased metabolism resulted decreased vesicular Aa levels, as elevated concentrations of its metabolic products (prostaglandin E2, PGE_2_; 12-hydroxyeicosatetraenoic acid, 12-HETE) were detected in malignant prostatic tissue [76, 77]. These studies highlight the significant role of EVs in Aa metabolism and PCa development.

In general, the potential of FAs, such as Aa to support cancer progression, has been reported in several previous papers [78–82]. For instance, Liu et al. have found increased serum levels of free fatty acids (FFA), Aa, linoleic acid (LA), and 15-HETE in lung adenocarcinoma patients. The group concluded that there is considerable basic evidence supporting the contribution of FFAs in tumor development and progression in lung cancer [83]. In PANC1 human pancreatic carcinoma cell-derived ELVs, the presence of 15-HETrE was shown and Pham et al. highlighted that this polyunsaturated FA participates in tumorigenesis and modulates Aa metabolism [84].

#### Sphingolipids, glycerophospholipids, and triacylglycerols

Several studies have investigated the lipid composition of cancer EVs. Altadill et al. have shown that ELVs isolated from the plasma of healthy controls or patients with endometrioid adenocarcinoma have significant amounts of glycerophospholipids (probably due to the exosomal membrane) and sphingolipids (29% of the total metabolite cargo of ELVs). They listed PI (16:0/22:4), PE (22:2/16:1), galactosylceramide (GalCer) (d18:2/16:0), glycerophosphocholine (GPCho) (18:0/14:0), or triacylglycerol (TG) (12:0/12:0/20:5) as the abundant lipids. In addition, the presence of phosphoglycerol (PG) (16:0/16:0), a precursor of cardiolipin, was validated and highlighted in plasma ELVs [44]. Cardiolipin is located in the inner membrane of mitochondria, and its concentration and distribution changes in mitochondria were observed in several diseases, including cancer [85].

In the study of Altadill et al., the metabolome of PANC1 pancreatic cell line-derived ELVs was also dominated by glycerophospholipids and sphingolipids with a proportion of 56% [44].

Lipids are sensitive biomarkers of pathophysiological changes. Significantly increased levels of several lysophosphatidylethanolamines (LPE), Cer, and PC were observed in MPE EVs compared to TPE vesicles [33]. Previous studies revealed that these lipids play a critical role in the immune response, cellular signaling, and proliferation. For instance, Kachler et al. found that Cers are related to metastasis and immune evasion in lung cancer [86]. Similarly, Luo et al. revealed an association between the levels of most PCs, PIs, and SMs and the clinical parameters of CEA and others [33]. CEA is an important tumor marker for colorectal and other carcinomas and plays a role in cell adhesion, signal transduction, and innate immunity [87]. The close relationships described by Luo et al. also suggest that the metabolites investigated are suitable for phenotypic characterization of MPE and TPE [33].

In the same study, more TGs were found in MPE EVs compared to EVs of TPE samples [33]. TGs are considered to be the main energy storage molecules, and elevated levels of TGs have also been observed in lung cancer tissues [88]. Additionally, 12 metabolites including PEs, DAGs, hexa-Cer, malic acid, and palmitic acid were elevated in MPE-lEVs. In general, more sphingolipids and glycerophospholipids were enriched in lEVs, while more FAs and glycolipids were enriched in sEVs. In addition, unique metabolic enrichment signatures were found both in TPE and MPE EVs providing the opportunity to track the unique biogenesis and function of the two EV subgroups in TPE and MPE [33].

DAGs are important messenger molecules in intercellular communication [89]. Nishida-Aoki et al. have shown that unsaturated DAGs are enriched in EVs from highly metastatic breast cancer. They also proved that the biological activity of the EVs to induce protein kinase D (PKD)/PKCµ phosphorylation in endothelial cells leads to neoangiogenesis. As DAG-mediated PKC activation occurs in many other cancer-related functions, such as cell proliferation and immune reactions, they concluded that DAG in cancer EVs may contribute to the EV-mediated education of the recipient cells to support tumor progression [90].

Clos-Garcia et al. found a selective decrease of Cers in urine EVs that correlates with PCa aggressiveness suggesting that Cers may have both cell-autonomous and non-cell-autonomous functions to limit cancer progression [63]. Kuc et al. showed that ceramide-1-phosphate (C1P) is a modulator of pancreatic cancer stem cell (PCSC) migration and fibronectin-specific based adhesion. They also identified pancreatic ductal adenocarcinoma (PDAC) cells as a source of C1P and concluded that C1P-containing EVs might recruit PCSCs to sustain tumor growth and C1P release could be a mechanism that facilitates tumor progression [91].

Kelleher et al*.* reported that PS-expressing EVs derived from ascites fluids and solid tumors of ovarian cancer patients induce a rapid and reversible arrest of the T cell receptor signaling in the CD4 + and CD8 + T cells through a DAG kinase-mediated inactivation of DAG. This finding offers therapeutic strategies, such as targeting PS-expressing EVs or the application of anti-PS antibodies or DAG kinase inhibitors (DGK*i*), which may enhance the patients’ T-cell responses to their tumor [92].

The lipid content of EVs has a crucial role in the adaptive response of tumors as well. Jung et al*.* found that phospholipid signatures of tumor EVs are related to gefitinib-resistance in non-small-cell lung cancer cells [93]. As a survival response to hypoxic stress, human PCa cells and EVs accumulate triglycerides, which support growth following reoxygenation [74].

#### Cholesterol and steroids

Cholesterol levels in EVs have been extensively studied using a wide range of experimental methods, and findings indicate that cholesterol is essential for the biogenesis, secretion, membrane stability and uptake of the vesicles as well [94]. As cholesterol is involved in the entire journey of EVs, it has a fundamental role in the EV-mediated signaling as well.

The human SOJ-6 pancreatic tumor cell-derived exosomes were shown to induce (glyco)protein ligand-independent apoptosis and inhibit the Notch-1 pathway in differentiated carcinoma cells, which indirectly favors the growth of undifferentiated tumor cells [95]. Beloribi et al. hypothesized that SOJ-6 exosomes interacted with tumor cells through cholesterol-rich membrane microdomains and exosomal lipids were the key elements to induce apoptosis. Through designing Synthetic Exosome-Like Nanoparticles (SELN) based on the lipid composition of SOJ-6 exosomes enriched in cholesterol and SM and depleted in phospholipids, they proved the role of lipids (i) in the interaction of SELNs and tumor cells and (ii) in induced cell death with inhibition of the Notch-1 pathway [96].

Clos-Garcia et al*.* detected an elevated level of dehydroepiandrosterone sulfate (DHEAS), an intermediary metabolite of androgen synthesis, in PCa urinary EVs, which suggests a potential role for EVs in androgen signaling in neighboring or distal cells [63].

### Carbohydrates, carbonic acids

#### Carbohydrates

Tumor cells possess an extraordinary capacity to regulate their energy metabolism as part of their tumor survival strategies [97]. One of the primary metabolic features of tumor cells is the Warburg effect, also known as aerobic glycolysis, which is characterized by an elevated rate of glycolysis even in the presence of oxygen. A large amount of glycolytic intermediates might be used to satisfy the metabolic requirements of proliferating cells [98].

Puhka et al*.* studied the metabolic profile of platelet- and urinary-derived EVs from PCa patients, and in both EV samples they observed a high concentration of D-ribose 5-phosphate, which is a major product of the cytosolic pentose-phosphate pathway and a key precursor for NAD^+^ and nucleotide biosynthesis [59]. Additionally, not only the D-ribose 5-phosphate concentration was increased in EVs, but also enzymes related to the pentose-phosphate pathway, such as glucose-6-phosphate dehydrogenase, transketolase, and transaldolase [99]. Numerous studies have demonstrated that the pentose-phosphate pathway serves an essential role for a cancer cell growth regulation and that the enzymes and metabolites delivered by EVs may contribute to the intense proliferation and cancer progression [100].

Furthermore, Wojakowska and colleagues studied the metabolic profiles of serum and serum-derived exosomes in HNC patients. Forty-six metabolites were identified in serum-derived exosome samples, including levoglucosan and 2,3-diphosphoglyceric acid. Metabolites that were detected in cancer but not in control samples were associated with energy metabolism [45].

#### Carbonic acids

Vallabhaneni et al. [61] found lactic acid in EVs secreted by mesenchymal stem/stromal cells from patients. The presence of lactic acid in the TME was shown to be linked to the improved capacity of tumor cells to withdraw hypoxic and nutrient-deprived core environments. Moreover, low pH caused by lactic acid is a known strategy of cancer cells to evade immune surveillance [101]. It is also worth mentioning that low pH, which is one of the hallmarks of cancer, enhanced exosome release and uptake in a melanoma cell line model [102].

Oncometabolites are common cellular metabolites that show abnormal accumulation in malignancies in comparison to non-proliferating cells and possess pro-oncogenic properties. These compounds are the products of cancer cell gene mutations or hypoxia-driven enzyme promiscuity. Accumulation of these oncometabolites in cancer cells results in metabolic and epigenetic changes, post-translational modifications, and other tumor-promoting effects [103]. Succinate, D-2-hydroxyglutarate (D-2-HG), L-2-hydroxyglutarate (L-2-HG), and fumarate are the four oncometabolites identified so far. All four oncometabolites are produced in the mitochondria (during TCA cycle) and can induce comparable changes in cancer cells, such as hypermethylation and pseudohypoxia, which results in metabolic and epigenetic changes, post-translational modifications and other tumorigenic characteristics [48].

Succinate, together with the three other oncometabolites, is a small molecule that accumulates in cancer cells as a result of gain-of-function or loss-of-function mutations in genes encoding energy metabolism enzymes. Elevated levels of succinate were measured in EVs from prostate, CTCL, and CC cell lines (PC-3, Mac-2A, RKO) compared to their respective control EVs (PNT2, PBMC, CCD841) [48]. In addition, succinate promotes tumorigenesis through a number of ways, such as generating epigenetic modifications and increasing cancer cell angiogenesis, invasion, and migration [104, 105]. Elevated levels of succinate levels have been found in cancer tissues, and biofluids of patients with various malignancies, including prostate and colorectal cancer [106], and hepatocarcinoma.

TCA cycle intermediates such as succinate, fumarate, and L-2-HG can alter the response of both the innate and adaptive immune systems. Through inhibition of histone and DNA demethylases, 2-HG and fumarate can also alter the epigenetic landscape of cells [107]. Endogenous fumarate was reported to suppress GAPDH via succination in macrophages [108]. Succinate, fumarate, and L-2-HG can inhibit prolyl-hydroxylases (PHDs) in normoxic environments leading to a pseudohypoxic state [109]. Inhibition of PHD enzymes results in stabilization of HIFs [110]. The HIF system plays a critical role in the regulation of a broad range of cellular and systemic responses to hypoxia. Thus, HIF-mediated pathways affect metabolic adaptation by increasing glucose uptake, lactate production, while decreasing respiration. HIF1α is a key regulator of EV production under hypoxia [111, 112].

Zhao et al*.* reported elevated lactate and acetate levels in both prostate and pancreatic CDEs [58]. Moreover, investigation of intra-exosomal metabolites revealed high citrate and pyruvate concentrations, as well as the significant presence of α-ketoglutarate, fumarate, and malate. These metabolites together with others – such as AAs – can replenish TCA cycle metabolites, and act as a source for lipid biosynthesis. Pyruvate is converted to acetyl-CoA by mitochondrial pyruvate dehydrogenase (PDH), while acetate is transferred into cells and transformed to acetyl-CoA through acetyl-CoA synthase [113–116]. Acetyl-CoA is the first step in lipid biosynthesis, which helps proliferating cells meet their biosynthetic needs. Recent findings implicate that exosomes of the TME can participate in the induced metabolic rewiring in cancer cells [117–119].

### Adenosine and other purine metabolites

Extracellular adenosine can be produced by cells, or it can be generated from extracellular ATP. Adenosine has an extremely short half-life (10 s) in the extracellular environment due to its quick uptake by cells and irreversible conversion to inosine [120, 121]. Considerable research has been conducted in the last few years on the diverse roles and associated mechanisms of extracellular adenosine signaling. Extracellular adenosine has a wide variety of effects on cell cycle control, immunoregulation, and cytokine regulation via both direct and indirect processes, eventually contributing to the development of malignant diseases [122]. Adenosine has been detected in urinary EVs from prostate cancer patients [59]. Sayner et al*.* have demonstrated that EVs encapsulate cAMP to offer a second messenger compartment [123]. Ludwig et al. have recently reported that exosomes from HNC squamous cell carcinoma (HNSCC) cell lines contain cAMP and adenosine as well as adenosine metabolites, i.e. inosine, hypoxanthine and xanthine. This exosomal repository of adenosine and inosine provides a unique and key pathway for the distal transport of these purines. By shielding them from the quick uptake and metabolism, exosomes can shuttle adenosine and inosine across cells, tissues, and organ systems. Ludwig and colleagues demonstrated that exosomes from HNSCC culture supernatant contain a variety of purine metabolites, the most abundant of which are adenosine and inosine. Purine metabolites, including adenosine, were much more abundant in exosomes isolated from the plasma of HNSCC patients than in exosomes obtained from normal donors. Exosomes from patients with early-stage illness and no lymph node metastases had considerably higher levels of adenosine and 5'-GMP. At the same time, exosomal levels of purine metabolites were reduced in patients with advanced cancer and nodal involvement. Decreased purine concentration in circulating exosomes may be the result of purine metabolites being primarily used for cellular maintenance and proliferation in metastatic tumor cells instead of being packaged into exosomes and exported outside of the cell. This suggests that the molecular composition of tumor-derived exosomes and circulating exosomes is quantitatively and, possibly, qualitatively different in advanced stages compared to early malignancies [124].

Clayton and colleagues demonstrated that exosomes produced by several cancer cell types have a high capacity for ATP and 5’-AMP phosphohydrolysis, which is partly attributed to the exosomal expression of CD39 and CD73 [125]. Exosomes can carry out both hydrolytic steps sequentially to convert extracellular ATP to adenosine. Exosome-produced adenosine can induce a cAMP response in adenosine A_2A_ receptor-positive but not A_2A_ receptor-negative cells.

A recently discovered pathway, the adenosine A_2B_ receptor-mediated signaling for exosome-induced angiogenesis contributes to the reprogramming of endothelial cells (ECs) to an angiogenic phenotype by direct interaction, and also to the reprogramming of other cell types found in the TME, such as macrophages [126]. As previously described, A_2B_ receptor stimulates the growth of ECs, induces angiogenesis, leads to VEGF production and upregulation of eNOS in ECs, and stimulates macrophages to release pro-angiogenic factors [127].

Tadokoro et al*.* showed another mechanism leading to an increase in extracellular adenosine levels, in which perforin secreted by CD8 + cytotoxic T cells disrupts the membrane of breast adenocarcinoma-derived EVs, and adenosine passively diffuses out. Adenosine from EVs acts as an immunosuppressive metabolite by binding to the adenosine receptor and inhibits perforin secretion by cytotoxic T lymphocytes [128].

Hypoxanthine, a purine derivative, is a potential intermediate in the metabolism of adenosine and also in the synthesis of nucleic acids. Glyceraldehyde 3-phosphate (G-3-P) is an intermediate in glycolysis. Luo and colleagues detected elevated levels of hypoxanthine and G-3-P in the lEVs in the MPE in comparison to lEVs of TPE [33], which may indicate accelerated glycolysis and nucleic acid formation.

However, Ronquist et al. reported that human seminal prostasomes contain glycolytic enzymes [129]. They detected the full set of glycolytic enzymes in PCa cell-derived exosomes and observed that both types of vesicles were capable of producing ATP when substrates were available [130]. Moreover, they reported a marked distinction between the high ATPase activity of prostasomes and the low ATPase activity of a malignant cell (PC3)-derived exosomes, which leads to a larger net gain of ATP in these latter exosomes. In contrast to prostasomes, the net ATP gain of metastatic PCa cell line exosomes was considerable due to their downregulated ATPase activity. This group also found that normal and prostate cancer cells uptake EVs (prostasomes and PC3 exosomes) in an energy-dependent manner. This uptake mechanism required a continuous glycolytic flux and extracellular ATP production by EVs and/or intracellularly by recipient cells in conjunction with the presence of a functioning vacuolar-type H( +)-ATPase (V-ATPase) [130].

### Other metabolites

Few additional compounds in HNC serum-derived EVs were found to be markedly downregulated compared to healthy controls. These include citric acid, 4-hydroxybenzoic acid, and propylene glycol, while 1-hexadecanol was markedly upregulated. A few other metabolites, 1,1-dimethoxyheptane, oxoadipic acid, paramethadione could only be detected in EVs, but not in whole serum samples [45].

Folate (B9 vitamin) as a cancer-associated metabolite was identified in greater amounts in CTCL and in PCa cell line-derived EVs compared to control EVs [48]. Along with folate, overexpression of pantothenic acid (B5), niacin (B3), thiamine (B1), and pyridoxine (B6) may boost one-carbon metabolism directly or indirectly through their roles as coenzymes, hence promoting cancer development [131]. One-carbon units are required for nucleotide synthesis, methylation, and reductive metabolism, all of which contribute to the rapid proliferation of cancer cells. Also in cell line-derived EVs from PCa and CTLC, Palviainen et al*.* have detected elevated levels of creatinine [48].

### Pathway analysis of EV metabolites

**Fig. 4:**
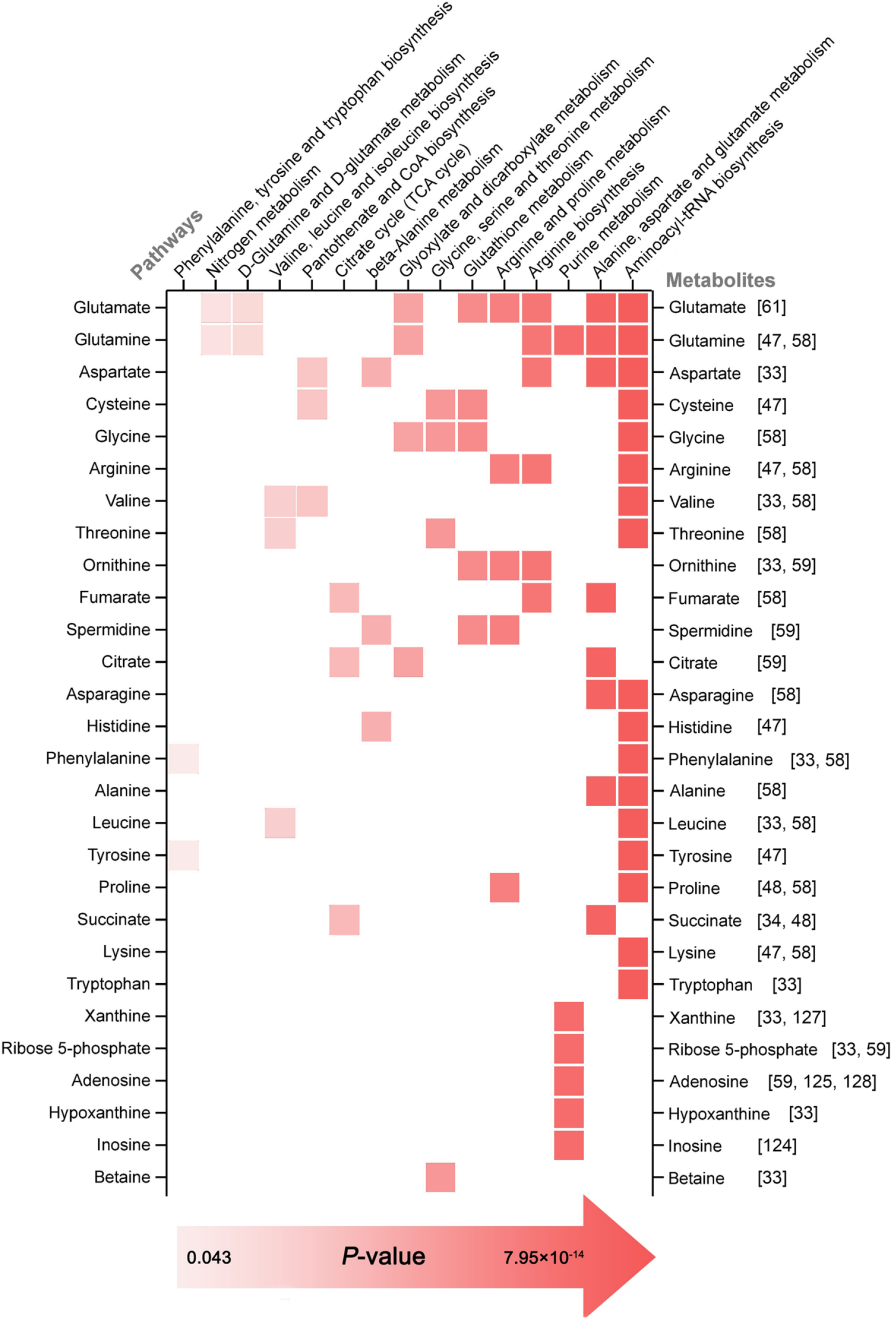
A matrix representation of metabolites and their associated pathways. Shades of red indicate the *P* values, which refers to the significance level of the association with different pathways. The significance increases from left to right of the graph. From top to bottom, the number of pathways associated with a metabolite decreases. The figure was created using RAWGraphs and GIMP

In order to explore the relationship between metabolites found in the literature and the pathways potentially involved, we performed pathway analyses (Fig. 4). This involved a total of 62 metabolites obtained from the processed literature, and 28 of these metabolites were strongly associated with 15 different pathways. These pathways are mainly those related to AAs, but significant associations were also found for glutathione, glyoxylate, dicarboxylate metabolism, TCA cycle, pantothenate and CoA biosynthesis.

In many cases, metabolites in the same column are subsequent steps in a metabolic pathway based on MetaboAnalyst. For example, in arginine biosynthesis glutamate is converted into ornithine in four steps, which in turn is converted into arginine in two steps (with the addition of aspartate). During the reaction, fumarate is released as a side product. The metabolism of glutathione serves as another example, in which glutathione is synthesized using cysteine, glutamate and glycine in subsequent steps, and then reacts with ornithine and spermidine to form trypanothione. With regard to carbohydrate metabolism, succinate, fumarate and citrate are associated with the TCA cycle, in which these molecules act as both substrates and products.

These results show a predominance of pathways associated with AA metabolism, but this analysis has serious limitations. For example, the metabolites from different sources were examined using different methods. The outcome of the pathway analysis is strongly influenced by the fact that most functional metabolomics studies have explicitly focused on AAs, resulting in a high number of AAs and their associated pathways. Nevertheless, the results suggest that non-AA metabolites transported by EVs may also play a role in amino metabolism. In addition, the results reveal that certain metabolites, such as glutamine/glutamate, may exert a wide range of effects on the metabolism of recipient cells entering a number of pathways.

## Conclusion and future directions

The stroma is a dynamic environment that is constantly evolving. Tumor-stroma interactions alter the microenvironment, making it more permissive towards cancer cells [132, 133]. Throughout the course of carcinogenesis, tumor cell hierarchies and various cellular components in the microenvironment co-evolve [134].

Understanding how cancer cells interact with the TME is critical for designing medicines that can halt tumor development and spread. Several studies have demonstrated that sEVs can facilitate communication between cancer cells and stromal cells inside the TME [135, 136]. EVs have emerged as a crucial mode of communication between different cell types in the TME. EVs transfer information across cells and reprogram the recipients [23, 24, 137]. In other words, current research indicates that EVs have the capacity to influence recipient cell proliferation, survival, and immune effector status [25–28].

It is also important to note that cancer changes cell metabolism. Typical metabolic conditions in the TME include hypoxia, starvation, and acidosis [133, 138]. The metabolic rewiring alters the secretion rate and metabolite content of cancer-derived EVs as well, but this cargo remains poorly characterized. Most of the current studies focus on the analysis of RNA [139, 140], or protein profiles [31] of EVs and related effects, and little is known about their metabolite cargo and function in recipient cells, which makes this field a fresh area of vesicle research.

Metabolites of EVs from human body fluids represent a goldmine of tumor biomarkers. However, the lack of a consensual approach for the separation of EVs is one a major obstacle to the advancement of EV research. In addition, the metabolite profile of EVs are potentially influenced by pre-analytical factors including external (storage, handling, analysis method) and internal (enzyme activity, sample contamination) factors. This may also account for the heterogeneity of experimental outcomes in EV research, since single or multiple EV subtypes with varying compositions and purities reveal method-dependent EV content and function. Given the wide range of constantly evolving EV isolation techniques, analysis methods, and applications available, MISEV2018 was unable to provide standard protocol recommendations yet. In our conclusion, there is not a gold standard method, which is optimal for all sample types and volumes, EV subtypes, and budgets. The use of EV-TRACK [141] may help choose the most advantageous method for EVs isolation and characterization.

There are several gaps in the knowledge on the effects of metabolites from cancer EVs. This review aimed to collect the current results on the biological activity of tumor EV metabolites (Fig. 3, Fig. 4). Innovative metabolomics technology, methods and their applications in clinical pharmacology have made significant progress during the last few years. The study of EV metabolites has shown remarkable potential and provides a new perspective in understanding cancer progression. Once methodologies are standardized, this knowledge will serve as a novel tool to identify new diagnostic biomarkers of cancer, to explain pathological mechanisms, to find possible therapeutic targets, to predict the biochemical and physiological effects of therapies, and to aid following up treatments of cancer.

It is important to note that investigating one or a few molecules in a subgroup of EVs allows only a partial insight into the functional role of the studied EV population. In contrast, analysis of the whole EV molecular pattern of the whole EV set would provide more relevant results. This approach has recently been applied in clinical studies for tumor diagnostic purposes [142–144].

## Methodology

This review is based on 95 articles related to the metabolomic research of EVs. The publications on the metabolomics of tumor EVs were selected based on the following criteria: (a) any type of EVs were investigated, (b) EVs were isolated by any method, (c) source of EVs were either* ex vivo* or *in vitro* samples, (d) *ex vivo* samples were tumor tisssues, CAFs, stromal cells or different body fluids of cancer patients (e) *in vitro* samples were tumor cell lines, (f) methods and equipment used in metabolomics studies were not necessarily identical across the studies. In some studies, metabolomic analyses were not accompanied by functional assays. In these cases, information on the potential tumor/metabolome-related effects of the molecule in question was gathered from other, non-EV-related research.

Based on the literature, pathway analyses were performed using MetaboAnalyst 5.0 software with the Kyoto Encyclopedia of Genes and Genomes database. Pathway enrichment and pathway topology were determined by hypergeometric test and relative-betweenness centrality method. Figures were created using RawGraphs 2.0 and CytoScape 3.9.

## Abbreviations

**AA**: Amino acid
**Aa**: Arachidonic acid
**ATP**: Adenosine triphosphate
**C1P**: Ceramide-1-phosphate
**CAF**: Cancer-associated fibroblast
**CD81sEV**: CD81-expressing small extracellular vesicle
**CDE**: CAF exosome
**CEA**: Carcinoembryonic antigen
**Cer**: Ceramide
**CTCL**: Cutaneous T-cell lymphoma
**D-2-HG**: D-2-hydroxyglutarate
**DAG**: Diacylglycerol
**EC**: Endothelial cell
**ELV**: Exosome-like vesicle
**EV**: Extracellular vesicle
**FA**: Fatty acid
**FFA**: Free fatty acid
**HIF-1α**: Hypoxia-inducible factor-α
**HNC**: Head and neck cancer
**HNSCC**: HNC squamous cell carcinoma
**ISEV**: International Society for Extracellular Vesicles
**lEV**: Large extracellular vesicle
**LPE**: Lysophosphatidylethanolamine
**MISEV**: Minimal Information for Studies of Extracellular Vesicles
**MPE**: Malignancy pleural effusion
**MVB**: Multivesicular body
**PC**: Phosphatidylcholine
**PCa**: Prostate cancer
**PE**: Phosphatidylethanolamine
**PG**: Phosphoglycerol
**PI**: Phosphatidylinositol
**PS**: Phosphatidylserine
**sEV**: Small extracellular vesicle
**SM**: Sphingomyelin
**TCA**: Tricarboxylic acid cycle
**TG**: Triacylglycerol
**TME**: Tumor microenvironment
**TPE**: Tuberculosis pleural effusion

## References

[1] Théry C, Witwer KW, Aikawa E, Alcaraz MJ, Anderson JD, Andriantsitohaina R, Antoniou A, Arab T, Archer F, Atkin-Smith GK, Ayre DC, Bach JM, Bachurski D, Baharvand H, Balaj L, Baldacchino S, Bauer NN, Baxter AA, Bebawy M, Beckham C, Zuba-Surma EK (2018). Minimal information for studies of extracellular vesicles 2018 (MISEV2018): A position statement of the International Society for Extracellular Vesicles and update of the MISEV2014 guidelines. Journal of extracellular vesicles.

[2] Raposo G, Stahl PD (2019). Extracellular vesicles: A new communication paradigm?. Nature reviews. Molecular cell biology.

[3] Hessvik NP, Llorente A (2018). Current knowledge on exosome biogenesis and release. Cellular and molecular life sciences : CMLS.

[4] Bebelman MP, Smit MJ, Pegtel DM, Baglio SR (2018). Biogenesis and function of extracellular vesicles in cancer. Pharmacology & Therapeutics.

[5] van Niel, G. & Théry C. (2020). Extracellular vesicles: eat glutamine and spit acidic bubbles. *The EMBO Journal,* 39(16):e105119. 10.15252/embj.2020105119 10.15252/embj.2020105119 PMC7429734 32809264

[6] Simeone P, Bologna G, Lanuti P, Pierdomenico L, Guagnano MT, Pieragostino D, Del Boccio P, Vergara D, Marchisio M, Miscia S, Mariani-Costantini R (2020). Extracellular Vesicles as Signaling Mediators and Disease Biomarkers across Biological Barriers. International journal of molecular sciences.

[7] Tao SC, Guo SC (2020). Role of extracellular vesicles in tumour microenvironment. Cell communication and signaling : CCS.

[8] Kalluri, R., & LeBleu, V. S. (2020). The biology**,** function**,** and biomedical applications of exosomes. *Science (New York, N.Y.)*, *367*(6478), eaau6977. 10.1126/science.aau6977 10.1126/science.aau6977 PMC7717626 32029601

[9] Möller A, Lobb RJ (2020). The evolving translational potential of small extracellular vesicles in cancer. Nature reviews. Cancer.

[10] Brennan K, Martin K, FitzGerald SP, O’Sullivan J, Wu Y, Blanco A, Richardson C, Mc Gee MM (2020). A comparison of methods for the isolation and separation of extracellular vesicles from protein and lipid particles in human serum. Scientific Reports.

[11] Sun, L., & Meckes, D. G., Jr. (2018). Methodological approaches to study extracellular vesicle miRNAs in Epstein−Barr virus-associated cancers. *International Journal of Molecular Sciences*, *19*(9). 10.3390/ijms19092810 10.3390/ijms19092810 PMC6164614 30231493

[12] Mathieu M, Martin-Jaular L, Lavieu G, Théry C (2019). Specificities of secretion and uptake of exosomes and other extracellular vesicles for cell-to-cell communication. Nature cell biology.

[13] Colombo M, Raposo G, Théry C (2014). Biogenesis, Secretion, and Intercellular Interactions of Exosomes and Other Extracellular Vesicles. Annual Review of Cell and Developmental Biology.

[14] van Niel G, D'Angelo G, Raposo G (2018). Shedding light on the cell biology of extracellular vesicles. Nature reviews. Molecular cell biology.

[15] Harmati M, Gyukity-Sebestyen E, Dobra G, Janovak L, Dekany I, Saydam O, Hunyadi-Gulyas E, Nagy I, Farkas A, Pankotai T, Ujfaludi Z, Horvath P, Piccinini F, Kovacs M, Biro T, Buzas K (2019). Small extracellular vesicles convey the stress-induced adaptive responses of melanoma cells. Scientific reports.

[16] Harmati M, Tarnai Z, Decsi G, Kormondi S, Szegletes Z, Janovak L, Dekany I, Saydam O, Gyukity-Sebestyen E, Dobra G, Nagy I, Nagy K, Buzas K (2017). Stressors alter intercellular communication and exosome profile of nasopharyngeal carcinoma cells. Journal of oral pathology & medicine : Official publication of the International Association of Oral Pathologists and the American Academy of Oral Pathology.

[17] Herrmann IK, Wood M, Fuhrmann G (2021). Extracellular vesicles as a next-generation drug delivery platform. Nature nanotechnology.

[18] Mulcahy, L. A., Pink, R. C., & Carter, D. R. (2014). Routes and mechanisms of extracellular vesicle uptake. Journal of extracellular vesicles 3. 10.3402/jev.v3.24641 10.3402/jev.v3.24641 PMC4122821 25143819

[19] French KC, Antonyak MA, Cerione RA (2017). Extracellular vesicle docking at the cellular port: Extracellular vesicle binding and uptake. Seminars in cell & developmental biology.

[20] Witwer KW, Théry C (2019). Extracellular vesicles or exosomes? On primacy, precision, and popularity influencing a choice of nomenclature. Journal of extracellular vesicles.

[21] Johnsen, K. B., Gudbergsson, J. M., Andresen, T. L., & Simonsen, J. B. (2019). What is the blood concentration of extracellular vesicles? Implications for the use of extracellular vesicles as blood-borne biomarkers of cancer. *Biochimica et biophysica acta. Reviews on cancer*, *1871*(1), 109–116. 10.1016/j.bbcan.2018.11.006 10.1016/j.bbcan.2018.11.006 30528756

[22] Cappello F, Logozzi M, Campanella C, Bavisotto CC, Marcilla A, Properzi F, Fais S (2017). Exosome levels in human body fluids: A tumor marker by themselves?. European journal of pharmaceutical sciences : Official journal of the European Federation for Pharmaceutical Sciences.

[23] Yang E, Wang X, Gong Z, Yu M, Wu H, Zhang D (2020). Exosome-mediated metabolic reprogramming: The emerging role in tumor microenvironment remodeling and its influence on cancer progression. Signal transduction and targeted therapy.

[24] Parayath NN, Padmakumar S, Amiji MM (2020). Extracellular vesicle-mediated nucleic acid transfer and reprogramming in the tumor microenvironment. Cancer letters.

[25] Gulei D, Petrut B, Tigu AB, Onaciu A, Fischer-Fodor E, Atanasov AG, Ionescu C, Berindan-Neagoe I (2018). Exosomes at a glance - common nominators for cancer hallmarks and novel diagnosis tools. Critical reviews in biochemistry and molecular biology.

[26] Nogués L, Benito-Martin A, Hergueta-Redondo M, Peinado H (2018). The influence of tumour-derived extracellular vesicles on local and distal metastatic dissemination. Molecular aspects of medicine.

[27] Marar C, Starich B, Wirtz D (2021). Extracellular vesicles in immunomodulation and tumor progression. Nature immunology.

[28] Kahlert C, Kalluri R (2013). Exosomes in tumor microenvironment influence cancer progression and metastasis. Journal of molecular medicine (Berlin, Germany).

[29] Maia J, Caja S, Strano Moraes MC, Couto N, Costa-Silva B (2018). Exosome-Based Cell-Cell Communication in the Tumor Microenvironment. Frontiers in cell and developmental biology.

[30] Kucharzewska P, Christianson HC, Welch JE, Svensson KJ, Fredlund E, Ringnér M, Mörgelin M, Bourseau-Guilmain E, Bengzon J, Belting M (2013). Exosomes reflect the hypoxic status of glioma cells and mediate hypoxia-dependent activation of vascular cells during tumor development. Proceedings of the National Academy of Sciences of the United States of America.

[31] Haraszti RA, Didiot M-C, Sapp E, Leszyk J, Shaffer SA, Rockwell HE, Gao F, Narain NR, DiFiglia M, Kiebish MA, Aronin N, Khvorova A (2016). High-resolution proteomic and lipidomic analysis of exosomes and microvesicles from different cell sources. Journal of Extracellular Vesicles.

[32] Xu, R., Greening, D. W., Rai, A., Ji, H., & Simpson, R. J. (2015). Highly-purified exosomes and shed microvesicles isolated from the human colon cancer cell line LIM1863 by sequential centrifugal ultrafiltration are biochemically and functionally distinct. *Methods (San Diego, Calif.)*, *87*, 11–25. 10.1016/j.ymeth.2015.04.008 10.1016/j.ymeth.2015.04.008 25890246

[33] Luo P, Mao K, Xu J, Wu F, Wang X, Wang S, Zhou M, Duan L, Tan Q, Ma G, Yang G, Du R, Huang H, Huang Q, Li Y, Guo M, Jin Y (2020). Metabolic characteristics of large and small extracellular vesicles from pleural effusion reveal biomarker candidates for the diagnosis of tuberculosis and malignancy. Journal of extracellular vesicles.

[34] Palviainen M, Saari H, Kärkkäinen O, Pekkinen J, Auriola S, Yliperttula M, Puhka M, Hanhineva K, Siljander PR-M (2019). Metabolic signature of extracellular vesicles depends on the cell culture conditions. Journal of Extracellular Vesicles.

[35] Petucci C, Zelenin A, Culver JA, Gabriel M, Kirkbride K, Christison TT, Gardell SJ (2016). Use of Ion Chromatography/Mass Spectrometry for Targeted Metabolite Profiling of Polar Organic Acids. Analytical chemistry.

[36] Cui L, Liu J, Yan X, Hu S (2017). Identification of Metabolite Biomarkers for Gout Using Capillary Ion Chromatography with Mass Spectrometry. Analytical chemistry.

[37] Hayasaka R, Tabata S, Hasebe M, Ikeda S, Ohnuma S, Mori M, Soga T, Tomita M, Hirayama A (2021). Metabolomic Analysis of Small Extracellular Vesicles Derived from Pancreatic Cancer Cells Cultured under Normoxia and Hypoxia. Metabolites.

[38] Williams C, Palviainen M, Reichardt NC, Siljander PR, Falcón-Pérez JM (2019). Metabolomics Applied to the Study of Extracellular Vesicles. Metabolites.

[39] Pathan M, Fonseka P, Chitti SV, Kang T, Sanwlani R, Van Deun J, Hendrix A, Mathivanan S (2019). Vesiclepedia 2019: A compendium of RNA, proteins, lipids and metabolites in extracellular vesicles. Nucleic acids research.

[40] Keerthikumar S, Chisanga D, Ariyaratne D, Al Saffar H, Anand S, Zhao K, Samuel M, Pathan M, Jois M, Chilamkurti N, Gangoda L, Mathivanan S (2016). ExoCarta: A Web-Based Compendium of Exosomal Cargo. Journal of molecular biology.

[41] Kim, D. K., Kang, B., Kim, O. Y., Choi, D. S., Lee, J., Kim, S. R., Go, G., Yoon, Y. J., Kim, J. H., Jang, S. C., Park, K. S., Choi, E. J., Kim, K. P., Desiderio, D. M., Kim, Y. K., Lötvall, J., Hwang, D., & Gho, Y. S. (2013). EVpedia: an integrated database of high-throughput data for systemic analyses of extracellular vesicles. Journal of extracellular vesicles* 2,*10.3402/jev.v2i0.20384. 10.3402/jev.v2i0.20384 10.3402/jev.v2i0.20384 PMC3760654 24009897

[42] Hildebrandt A, Kirchner B, Nolte-'t Hoen E, Pfaffl M (2021). miREV: An Online Database and Tool to Uncover Potential Reference RNAs and Biomarkers in Small-RNA Sequencing Data Sets from Extracellular Vesicles Enriched Samples. Journal Of Molecular Biology.

[43] Hannafon BN, Ding WQ (2013). Intercellular communication by exosome-derived microRNAs in cancer. International Journal of Molecular Sciences.

[44] Altadill T, Campoy I, Lanau L, Gill K, Rigau M, Gil-Moreno A, Reventos J, Byers S, Colas E, Cheema AK (2016). Enabling Metabolomics Based Biomarker Discovery Studies Using Molecular Phenotyping of Exosome-Like Vesicles. PLoS ONE.

[45] Wojakowska A, Zebrowska A, Skowronek A, Rutkowski T, Polanski K, Widlak P, Marczak L, Pietrowska M (2020). Metabolic Profiles of Whole Serum and Serum-Derived Exosomes Are Different in Head and Neck Cancer Patients Treated by Radiotherapy. Journal of personalized medicine.

[46] Paolino G, Huber V, Camerini S, Casella M, Macone A, Bertuccini L, Iosi F, Moliterni E, Cecchetti S, Ruspantini I, Chiarotti F, Vergani E, Lalli L, Raggi C, Di Biase A, Calvieri S, Mercuri SR, Lugini L, Federici C (2021). The Fatty Acid and Protein Profiles of Circulating CD81-Positive Small Extracellular Vesicles Are Associated with Disease Stage in Melanoma Patients. Cancers.

[47] Onozato M, Tanaka Y, Arita M, Sakamoto T, Ichiba H, Sadamoto K, Kondo M, Fukushima T (2018). Amino acid analyses of the exosome-eluted fractions from human serum by HPLC with fluorescence detection. Practical laboratory medicine.

[48] Palviainen M, Laukkanen K, Tavukcuoglu Z, Velagapudi V, Kärkkäinen O, Hanhineva K, Auriola S, Ranki A, Siljander P (2020). Cancer Alters the Metabolic Fingerprint of Extracellular Vesicles. Cancers.

[49] Tanner JJ, Fendt S-M, Becker DF (2018). The Proline Cycle As a Potential Cancer Therapy Target. Biochemistry.

[50] Phang JM (2019). Proline Metabolism in Cell Regulation and Cancer Biology: Recent Advances and Hypotheses. Antioxidants & redox signaling.

[51] Huynh T, Zareba I, Baszanowska W, Lewoniewska S, Palka J (2020). Understanding the role of key amino acids in regulation of proline dehydrogenase/proline oxidase (prodh/pox)-dependent apoptosis/autophagy as an approach to targeted cancer therapy. Molecular and cellular biochemistry.

[52] Surazynski A, Donald SP, Cooper SK, Whiteside MA, Salnikow K, Liu Y, Phang JM (2008). Extracellular matrix and HIF-1 signaling: The role of prolidase. International journal of cancer.

[53] Maxwell PH, Eckardt KU (2016). HIF prolyl hydroxylase inhibitors for the treatment of renal anaemia and beyond. Nature reviews. Nephrology.

[54] Bellance N, Pabst L, Allen G, Rossignol R, Nagrath D (2012). Oncosecretomics coupled to bioenergetics identifies α-amino adipic acid, isoleucine and GABA as potential biomarkers of cancer: Differential expression of c-Myc, Oct1 and KLF4 coordinates metabolic changes. Biochimica et biophysica acta.

[55] Chen L, Miao Y, Liu M, Zeng Y, Gao Z, Peng D, Hu B, Li X, Zheng Y, Xue Y, Zuo Z, Xie Y, Ren J (2018). Pan-Cancer Analysis Reveals the Functional Importance of Protein Lysine Modification in Cancer Development. Frontiers in genetics.

[56] Takeda M, Takamiya A, Jiao JW, Cho KS, Trevino SG, Matsuda T, Chen DF (2008). alpha-Aminoadipate induces progenitor cell properties of Müller glia in adult mice. Investigative ophthalmology & visual science.

[57] Rosi A, Ricci-Vitiani L, Biffoni M, Grande S, Luciani AM, Palma A, Runci D, Cappellari M, De Maria R, Guidoni L, Pallini R, Viti V (2015). (1) H NMR spectroscopy of glioblastoma stem-like cells identifies alpha-aminoadipate as a marker of tumor aggressiveness. NMR in biomedicine.

[58] Zhao, H., Yang, L., Baddour, J., Achreja, A., Bernard, V., Moss, T., Marini, J. C., Tudawe, T., Seviour, E. G., San Lucas, F. A., Alvarez, H., Gupta, S., Maiti, S. N., Cooper, L., Peehl, D., Ram, P. T., Maitra, A., & Nagrath, D. (2016). Tumor microenvironment derived exosomes pleiotropically modulate cancer cell metabolism. *eLife*, *5*, e10250. 10.7554/eLife.10250 10.7554/eLife.10250 PMC4841778 26920219

[59] Puhka M, Takatalo M, Nordberg ME, Valkonen S, Nandania J, Aatonen M, Yliperttula M, Laitinen S, Velagapudi V, Mirtti T, Kallioniemi O, Rannikko A, Siljander PR, Af Hällström TM (2017). Metabolomic Profiling of Extracellular Vesicles and Alternative Normalization Methods Reveal Enriched Metabolites and Strategies to Study Prostate Cancer-Related Changes. Theranostics.

[60] Gökmen SS, Aygit AC, Ayhan MS, Yorulmaz F, Gülen S (2001). Significance of arginase and ornithine in malignant tumors of the human skin. The Journal of laboratory and clinical medicine.

[61] Vallabhaneni, K. C., Penfornis, P., Dhule, S., Guillonneau, F., Adams, K. V., Mo, Y. Y., Xu, R., Liu, Y., Watabe, K., Vemuri, M. C., & Pochampally, R. (2015). Extracellular vesicles from bone marrow mesenchymal stem/stromal cells transport tumor regulatory microRNA, proteins, and metabolites. *Oncotarget*, *6*(7), 4953–4967. 10.18632/oncotarget.3211 10.18632/oncotarget.3211 PMC4467126 25669974

[62] Soda K (2011). The mechanisms by which polyamines accelerate tumor spread. Journal of experimental & clinical cancer research : CR.

[63] Clos-Garcia M, Loizaga-Iriarte A, Zuñiga-Garcia P, Sánchez-Mosquera P, Rosa Cortazar A, González E, Torrano V, Alonso C, Pérez-Cormenzana M, Ugalde-Olano A, Lacasa-Viscasillas I, Castro A, Royo F, Unda M, Carracedo A, Falcón-Pérez JM (2018). Metabolic alterations in urine extracellular vesicles are associated to prostate cancer pathogenesis and progression. Journal of extracellular vesicles.

[64] Ho WS (2010). Angiogenesis: A new physiological role for N-arachidonoyl serine and GPR55?. British journal of pharmacology.

[65] Muro E, Atilla-Gokcumen GE, Eggert US (2014). Lipids in cell biology: How can we understand them better?. Molecular biology of the cell.

[66] Pizzinat N, Ong-Meang V, Bourgailh-Tortosa F, Blanzat M, Perquis L, Cussac D, Parini A, Poinsot V (2020). Extracellular vesicles of MSCs and cardiomyoblasts are vehicles for lipid mediators. Biochimie.

[67] Choi DS, Kim DK, Kim YK, Gho YS (2013). Proteomics, transcriptomics and lipidomics of exosomes and ectosomes. Proteomics.

[68] Skotland T, Sandvig K, Llorente A (2017). Lipids in exosomes: Current knowledge and the way forward. Progress in lipid research.

[69] Trajkovic, K., Hsu, C., Chiantia, S., Rajendran, L., Wenzel, D., Wieland, F., Schwille, P., Brügger, B., & Simons, M. (2008). Ceramide triggers budding of exosome vesicles into multivesicular endosomes. *Science (New York, N.Y.)*, *319*(5867), 1244–1247. 10.1126/science.1153124 10.1126/science.1153124 18309083

[70] Hannun YA, Obeid LM (2008). Principles of bioactive lipid signalling: Lessons from sphingolipids. Nature reviews. Molecular cell biology.

[71] Hashimoto, M., & Hossain, S. (2018). Fatty Acids: From Membrane Ingredients to Signaling Molecules. In Biochemistry and Health Benefits of Fatty Acids. *IntechOpen*. 10.5772/intechopen.80430

[72] Chen M, Huang J (2019). The expanded role of fatty acid metabolism in cancer: New aspects and targets. Precision clinical medicine.

[73] Padanad MS, Konstantinidou G, Venkateswaran N, Melegari M, Rindhe S, Mitsche M, Yang C, Batten K, Huffman KE, Liu J, Tang X, Rodriguez-Canales J, Kalhor N, Shay JW, Minna JD, McDonald J, Wistuba II, DeBerardinis RJ, Scaglioni PP (2016). Fatty Acid Oxidation Mediated by Acyl-CoA Synthetase Long Chain 3 Is Required for Mutant KRAS Lung Tumorigenesis. Cell reports.

[74] Schlaepfer, I. R., Nambiar, D. K., Ramteke, A., Kumar, R., Dhar, D., Agarwal, C., Bergman, B., Graner, M., Maroni, P., Singh, R. P., Agarwal, R., & Deep, G. (2015). Hypoxia induces triglycerides accumulation in prostate cancer cells and extracellular vesicles supporting growth and invasiveness following reoxygenation. *Oncotarget*, *6*(26), 22836–22856. 10.18632/oncotarget.4479 10.18632/oncotarget.4479 PMC4673203 26087400

[75] Bogatcheva NV, Sergeeva MG, Dudek SM, Verin AD (2005). Arachidonic acid cascade in endothelial pathobiology. Microvascular research.

[76] Yang P, Cartwright CA, Li J, Wen S, Prokhorova IN, Shureiqi I, Kim J (2012). Arachidonic acid metabolism in human prostate cancer. International Journal of Oncology.

[77] Chaudry AA, Wahle KW, McClinton S, Moffat LE (1994). Arachidonic acid metabolism in benign and malignant prostatic tissue in vitro: Effects of fatty acids and cyclooxygenase inhibitors. International journal of cancer.

[78] Vinciguerra M, Carrozzino F, Peyrou M, Carlone S, Montesano R, Benelli R, Foti M (2009). Unsaturated fatty acids promote hepatoma proliferation and progression through downregulation of the tumor suppressor PTEN. Journal of hepatology.

[79] Wen ZH, Su YC, Lai PL, Zhang Y, Xu YF, Zhao A, Yao GY, Jia CH, Lin J, Xu S, Wang L, Wang XK, Liu AL, Jiang Y, Dai YF, Bai XC (2013). Critical role of arachidonic acid-activated mTOR signaling in breast carcinogenesis and angiogenesis. Oncogene.

[80] Zhang Y, He C, Qiu L, Wang Y, Zhang L, Qin X, Liu Y, Zhang D, Li Z (2014). Serum unsaturated free Fatty acids: Potential biomarkers for early detection and disease progression monitoring of non-small cell lung cancer. Journal of Cancer.

[81] Borin TF, Angara K, Rashid MH, Achyut BR, Arbab AS (2017). Arachidonic Acid Metabolite as a Novel Therapeutic Target in Breast Cancer Metastasis. International journal of molecular sciences.

[82] Blücher C, Stadler SC (2017). Obesity and Breast Cancer: Current Insights on the Role of Fatty Acids and Lipid Metabolism in Promoting Breast Cancer Growth and Progression. Frontiers in endocrinology.

[83] Liu J, Mazzone PJ, Cata JP, Kurz A, Bauer M, Mascha EJ, Sessler DI (2014). Serum free fatty acid biomarkers of lung cancer. Chest.

[84] Pham H, Banerjee T, Ziboh VA (2004). Suppression of cyclooxygenase-2 overexpression by 15S-hydroxyeicosatrienoic acid in androgen-dependent prostatic adenocarcinoma cells. International journal of cancer.

[85] Zeczycki TN, Whelan J, Hayden WT, Brown DA, Shaikh SR (2014). Increasing levels of cardiolipin differentially influence packing of phospholipids found in the mitochondrial inner membrane. Biochemical and biophysical research communications.

[86] Kachler K, Bailer M, Heim L, Schumacher F, Reichel M, Holzinger CD, Trump S, Mittler S, Monti J, Trufa DI, Rieker RJ, Hartmann A, Sirbu H, Kleuser B, Kornhuber J, Finotto S (2017). Enhanced Acid Sphingomyelinase Activity Drives Immune Evasion and Tumor Growth in Non-Small Cell Lung Carcinoma. Cancer research.

[87] Hammarström S (1999). The carcinoembryonic antigen (CEA) family: Structures, suggested functions and expression in normal and malignant tissues. Seminars in cancer biology.

[88] Eggers LF, Müller J, Marella C, Scholz V, Watz H, Kugler C, Rabe KF, Goldmann T, Schwudke D (2017). Lipidomes of lung cancer and tumour-free lung tissues reveal distinct molecular signatures for cancer differentiation, age, inflammation, and pulmonary emphysema. Scientific reports.

[89] Almena M, Mérida I (2011). Shaping up the membrane: Diacylglycerol coordinates spatial orientation of signaling. Trends in biochemical sciences.

[90] Nishida-Aoki N, Izumi Y, Takeda H, Takahashi M, Ochiya T, Bamba T (2020). Lipidomic Analysis of Cells and Extracellular Vesicles from High- and Low-Metastatic Triple-Negative Breast Cancer. Metabolites.

[91] Kuc N, Doermann A, Shirey C, Lee DD, Lowe CW, Awasthi N, Schwarz RE, Stahelin RV, Schwarz MA (2018). Pancreatic ductal adenocarcinoma cell secreted extracellular vesicles containing ceramide-1-phosphate promote pancreatic cancer stem cell motility. Biochemical pharmacology.

[92] Kelleher RJ, Balu-Iyer S, Loyall J, Sacca AJ, Shenoy GN, Peng P, Iyer V, Fathallah AM, Berenson CS, Wallace PK, Tario J, Odunsi K, Bankert RB (2015). Extracellular Vesicles Present in Human Ovarian Tumor Microenvironments Induce a Phosphatidylserine-Dependent Arrest in the T-cell Signaling Cascade. Cancer immunology research.

[93] Jung JH, Lee MY, Choi DY, Lee JW, You S, Lee KY, Kim J, Kim KP (2015). Phospholipids of tumor extracellular vesicles stratify gefitinib-resistant nonsmall cell lung cancer cells from gefitinib-sensitive cells. Proteomics.

[94] Pfrieger FW, Vitale N (2018). Cholesterol and the journey of extracellular vesicles. Journal of lipid research.

[95] Ristorcelli E, Beraud E, Mathieu S, Lombardo D, Verine A (2009). Essential role of Notch signaling in apoptosis of human pancreatic tumoral cells mediated by exosomal nanoparticles. International journal of cancer.

[96] Beloribi S, Ristorcelli E, Breuzard G, Silvy F, Bertrand-Michel J, Beraud E, Verine A, Lombardo D (2012). Exosomal lipids impact notch signaling and induce death of human pancreatic tumoral SOJ-6 cells. PLoS ONE.

[97] Hanahan D, Weinberg RA (2011). Hallmarks of cancer: The next generation. Cell.

[98] Yu, L., Chen, X., Wang, L., & Chen, S. (2016). The sweet trap in tumors: aerobic glycolysis and potential targets for therapy. *Oncotarget, 7*(25), 38908–38926. 10.18632/oncotarget.7676 10.18632/oncotarget.7676 PMC5122440 26918353

[99] Yi H, Zheng X, Song J, Shen R, Su Y, Lin D (2015). Exosomes mediated pentose phosphate pathway in ovarian cancer metastasis: A proteomics analysis. International journal of clinical and experimental pathology.

[100] Jin L, Zhou Y (2019). Crucial role of the pentose phosphate pathway in malignant tumors. Oncology letters.

[101] Wang JX, Choi S, Niu X, Kang N, Xue H, Killam J, Wang Y (2020). Lactic Acid and an Acidic Tumor Microenvironment suppress Anticancer Immunity. International journal of molecular sciences.

[102] Parolini I, Federici C, Raggi C, Lugini L, Palleschi S, De Milito A, Coscia C, Iessi E, Logozzi M, Molinari A, Colone M, Tatti M, Sargiacomo M, Fais S (2009). Microenvironmental pH is a key factor for exosome traffic in tumor cells. The Journal of biological chemistry.

[103] Beyoğlu D, Idle JR (2021). Metabolic Rewiring and the Characterization of Oncometabolites. Cancers.

[104] Moosavi B, Zhu XL, Yang WC, Yang GF (2020). Molecular pathogenesis of tumorigenesis caused by succinate dehydrogenase defect. European journal of cell biology.

[105] Mu, X., Zhao, T., Xu, C., Shi, W., Geng, B., Shen, J., Zhang, C., Pan, J., Yang, J., Hu, S., Lv, Y., Wen, H., & You, Q. (2017). Oncometabolite succinate promotes angiogenesis by upregulating VEGF expression through GPR91-mediated STAT3 and ERK activation. *Oncotarget*, *8*(8), 13174–13185. 10.18632/oncotarget.14485 10.18632/oncotarget.14485 PMC5355086 28061458

[106] Dalla Pozza E, Dando I, Pacchiana R, Liboi E, Scupoli MT, Donadelli M, Palmieri M (2020). Regulation of succinate dehydrogenase and role of succinate in cancer. Seminars in cell & developmental biology.

[107] Martínez-Reyes I, Chandel NS (2020). Mitochondrial TCA cycle metabolites control physiology and disease. Nature communications.

[108] O'Neill LA, Kishton RJ, Rathmell J (2016). A guide to immunometabolism for immunologists. Nature reviews. Immunology.

[109] Kaelin WG, Ratcliffe PJ (2008). Oxygen sensing by metazoans: The central role of the HIF hydroxylase pathway. Molecular cell.

[110] Isaacs JS, Jung YJ, Mole DR, Lee S, Torres-Cabala C, Chung YL, Merino M, Trepel J, Zbar B, Toro J, Ratcliffe PJ, Linehan WM, Neckers L (2005). HIF overexpression correlates with biallelic loss of fumarate hydratase in renal cancer: Novel role of fumarate in regulation of HIF stability. Cancer Cell.

[111] Choudhry H, Harris AL (2018). Advances in Hypoxia-Inducible Factor Biology. Cell metabolism.

[112] Zhang W, Zhou X, Yao Q, Liu Y, Zhang H, Dong Z (2017). HIF-1-mediated production of exosomes during hypoxia is protective in renal tubular cells. American journal of physiology. Renal physiology.

[113] De Schrijver E, Brusselmans K, Heyns W, Verhoeven G, Swinnen JV (2003). RNA interference-mediated silencing of the fatty acid synthase gene attenuates growth and induces morphological changes and apoptosis of LNCaP prostate cancer cells. Cancer research.

[114] Feron O (2009). Pyruvate into lactate and back: From the Warburg effect to symbiotic energy fuel exchange in cancer cells. Radiotherapy and Oncology.

[115] Koukourakis, M. I., Giatromanolaki, A., Sivridis, E., Gatter, K. C., Harris, A. L., & Tumor and Angiogenesis Research Group (2005). Pyruvate dehydrogenase and pyruvate dehydrogenase kinase expression in non small cell lung cancer and tumor-associated stroma. *Neoplasia (New York, N.Y.)*, *7*(1), 1–6. 10.1593/neo.04373 10.1593/neo.04373 PMC1490315 15736311

[116] Zaidi N, Swinnen JV, Smans K (2012). ATP-citrate lyase: A key player in cancer metabolism. Cancer research.

[117] Cairns RA, Harris IS, Mak TW (2011). Regulation of cancer cell metabolism. Nature reviews. Cancer.

[118] Fiaschi T, Chiarugi P (2012). Oxidative stress, tumor microenvironment, and metabolic reprogramming: A diabolic liaison. International journal of cell biology.

[119] Rattigan YI, Patel BB, Ackerstaff E, Sukenick G, Koutcher JA, Glod JW, Banerjee D (2012). Lactate is a mediator of metabolic cooperation between stromal carcinoma associated fibroblasts and glycolytic tumor cells in the tumor microenvironment. Experimental cell research.

[120] Möser GH, Schrader J, Deussen A (1989). Turnover of adenosine in plasma of human and dog blood. The American journal of physiology.

[121] Zsuga J, Erdei T, Szabó K, Lampe N, Papp C, Pinter A, Szentmiklosi AJ, Juhasz B, Szilvássy Z, Gesztelyi R (2017). Methodical Challenges and a Possible Resolution in the Assessment of Receptor Reserve for Adenosine, an Agonist with Short Half-Life. Molecules (Basel, Switzerland).

[122] Cai Y, Feng L, Wang X (2018). Targeting the tumor promoting effects of adenosine in chronic lymphocytic leukemia. Critical reviews in oncology/hematology.

[123] Sayner, S. L., Choi, C. S., Maulucci, M. E., Ramila, K. C., Zhou, C., Scruggs, A. K., Yarbrough, T., Blair, L. A., King, J. A., Seifert, R., Kaever, V., & Bauer, N. N. (2019). Extracellular vesicles: another compartment for the second messenger, cyclic adenosine monophosphate. *American journal of physiology. Lung cellular and molecular physiology*, *316*(4), L691–L700. 10.1152/ajplung.00282.2018 10.1152/ajplung.00282.2018 PMC6483015 30758991

[124] Ludwig N, Yerneni SS, Azambuja JH, Gillespie DG, Menshikova EV, Jackson EK, Whiteside TL (2020). Tumor-derived exosomes promote angiogenesis via adenosine A_2B_ receptor signaling. Angiogenesis.

[125] Clayton, A., Al-Taei, S., Webber, J., Mason, M. D., & Tabi, Z. (2011). Cancer exosomes express CD39 and CD73, which suppress T cells through adenosine production. *Journal of immunology (Baltimore, Md. : 1950)*, *187*(2), 676–683. 10.4049/jimmunol.1003884 10.4049/jimmunol.1003884 21677139

[126] Ludwig, N., Jackson, E. K., & Whiteside, T. L. (2020). Role of exosome-associated adenosine in promoting angiogenesis. *Vessel plus*, *4*, 8. 10.20517/2574-1209.2019.37 10.20517/2574-1209.2019.37 PMC7266301 32490359

[127] Ludwig N, Gillespie DG, Reichert TE, Jackson EK, Whiteside TL (2020). Purine Metabolites in Tumor-Derived Exosomes May Facilitate Immune Escape of Head and Neck Squamous Cell Carcinoma. Cancers.

[128] Tadokoro H, Hirayama A, Kudo R, Hasebe M, Yoshioka Y, Matsuzaki J, Yamamoto Y, Sugimoto M, Soga T, Ochiya T (2020). Adenosine leakage from perforin-burst extracellular vesicles inhibits perforin secretion by cytotoxic T-lymphocytes. PLoS ONE.

[129] Ronquist, K. G., Ek, B., Stavreus-Evers, A., Larsson, A., & Ronquist, G. (2013). Human prostasomes express glycolytic enzymes with capacity for ATP production. *American journal of physiology. Endocrinology and metabolism*, *304*(6), E576–E582. 10.1152/ajpendo.00511.2012 10.1152/ajpendo.00511.2012 23341497

[130] Ronquist KG, Sanchez C, Dubois L, Chioureas D, Fonseca P, Larsson A, Ullén A, Yachnin J, Ronquist G, Panaretakis T (2016). Energy-requiring uptake of prostasomes and PC3 cell-derived exosomes into non-malignant and malignant cells. Journal of extracellular vesicles.

[131] Newman AC, Maddocks O (2017). One-carbon metabolism in cancer. British journal of cancer.

[132] Quail DF, Joyce JA (2013). Microenvironmental regulation of tumor progression and metastasis. Nature medicine.

[133] Finger EC, Giaccia AJ (2010). Hypoxia, inflammation, and the tumor microenvironment in metastatic disease. Cancer metastasis reviews.

[134] Korkaya H, Liu S, Wicha MS (2011). Breast cancer stem cells, cytokine networks, and the tumor microenvironment. The Journal of clinical investigation.

[135] Peinado H, Alečković M, Lavotshkin S, Matei I, Costa-Silva B, Moreno-Bueno G, Hergueta-Redondo M, Williams C, García-Santos G, Ghajar C, Nitadori-Hoshino A, Hoffman C, Badal K, Garcia BA, Callahan MK, Yuan J, Martins VR, Skog J, Kaplan RN, Brady MS, Lyden D (2012). Melanoma exosomes educate bone marrow progenitor cells toward a pro-metastatic phenotype through MET. Nature medicine.

[136] Gyukity-Sebestyén E, Harmati M, Dobra G, Németh IB, Mihály J, Zvara Á, Hunyadi-Gulyás É, Katona R, Nagy I, Horváth P, Bálind Á, Szkalisity Á, Kovács M, Pankotai T, Borsos B, Erdélyi M, Szegletes Z, Veréb ZJ, Buzás EI, Kemény L, Buzás K (2019). Melanoma-Derived Exosomes Induce PD-1 Overexpression and Tumor Progression via Mesenchymal Stem Cell Oncogenic Reprogramming. Frontiers in immunology.

[137] Gangoda L, Boukouris S, Liem M, Kalra H, Mathivanan S (2015). Extracellular vesicles including exosomes are mediators of signal transduction: Are they protective or pathogenic?. Proteomics.

[138] Sebestyén A, Kopper L, Dankó T, Tímár J (2021). Hypoxia Signaling in Cancer: From Basics to Clinical Practice. Pathology oncology research : POR.

[139] Min L, Zhu S, Chen L, Liu X, Wei R, Zhao L, Yang Y, Zhang Z, Kong G, Li P, Zhang S (2019). Evaluation of circulating small extracellular vesicles derived miRNAs as biomarkers of early colon cancer: A comparison with plasma total miRNAs. Journal of extracellular vesicles.

[140] Lázaro-Ibáñez E, Lunavat TR, Jang SC, Escobedo-Lucea C, Oliver-De La Cruz J, Siljander P, Lötvall J, Yliperttula M (2017). Distinct prostate cancer-related mRNA cargo in extracellular vesicle subsets from prostate cell lines. BMC Cancer.

[141] Van Deun J (2017). EV-TRACK: Transparent reporting and centralizing knowledge in extracellular vesicle research. Nature methods..

[142] Bukva M, Dobra G, Gomez-Perez J, Koos K, Harmati M, Gyukity-Sebestyen E, Biro T, Jenei A, Kormondi S, Horvath P, Konya Z, Klekner A, Buzas K (2021). Raman Spectral Signatures of Serum-Derived Extracellular Vesicle-Enriched Isolates May Support the Diagnosis of CNS Tumors. Cancers.

[143] Guerrini L, Garcia-Rico E, O'Loghlen A, Giannini V, Alvarez-Puebla RA (2021). Surface-Enhanced Raman Scattering (SERS) Spectroscopy for Sensing and Characterization of Exosomes in Cancer Diagnosis. Cancers.

[144] Samoylenko A, Kögler M, Zhyvolozhnyi A, Makieieva O, Bart G, Andoh SS, Roussey M, Vainio SJ, Hiltunen J (2021). Time-gated Raman spectroscopy and proteomics analyses of hypoxic and normoxic renal carcinoma extracellular vesicles. Scientific reports.

